# Automatic Search Dense Connection Module for Super-Resolution

**DOI:** 10.3390/e24040489

**Published:** 2022-03-31

**Authors:** Huaijuan Zang, Guoan Cheng, Zhipeng Duan, Ying Zhao, Shu Zhan

**Affiliations:** Key Laboratory of Knowledge Engineering with Big Data, Ministry of Education, School of Computer Science and Information Engineering, Hefei University of Technology, Hefei 230601, China; zanghj@mail.hfut.edu.cn (H.Z.); guoan@mail.hfut.edu.cn (G.C.); zhipengduan@mail.hfut.edu.cn (Z.D.); yingzhao@mail.hfut.edu.cn (Y.Z.)

**Keywords:** single image super-resolution, neural architecture search, dense connection

## Abstract

The development of display technology has continuously increased the requirements for image resolution. However, the imaging systems of many cameras are limited by their physical conditions, and the image resolution is often restrictive. Recently, several models based on deep convolutional neural network (CNN) have gained significant performance for image super-resolution (SR), while extensive memory consumption and computation overhead hinder practical applications. For this purpose, we present a lightweight network that automatically searches dense connection (ASDCN) for image super-resolution (SR), which effectively reduces redundancy in dense connection and focuses on more valuable features. We employ neural architecture search (NAS) to model the searching of dense connections. Qualitative and quantitative experiments on five public datasets show that our derived model achieves superior performance over the state-of-the-art models.

## 1. Introduction

Since it is difficult for the current visual effects of images to meet people’s needs, single image super-resolution (SISR) [1] and its related technologies have attracted widespread attention [2]. SISR is a low-level computer vision task for reconstructing high-resolution images from low-resolution images. Due to the rapid development of deep convolutional neural networks (CNNs), deep CNN-based approaches have gained better reconstruction results against traditional methods in the field of SISR [3,4].

Super-resolution convolutional neural network (SRCNN) [5] was the pioneer of deep CNN in super-resolution (SR) problems, and an end-to-end nonlinear mapping through only a three-layer convolutional network was established. Since then, numerous CNN-based algorithms have emerged and made remarkable progress. Very deep convolutional networks super-resolution (VDSR) [6] deepen the network to 20 layers by exploiting residual learning [7], which alleviated the training difficulty. Deep recursive residual network (DRRN) [8] equipped recursive blocks to obtain promising results with deeper network structures. However, these methods used the interpolated low-resolution (LR) images as input in the network, which undoubtedly led to computational and time burdens. Shi et al. [9] devised an efficient sub-pixel convolution to tackle this problem, which directly extracted feature maps from the LR images. Subsequently, Lim et al. [10] extended the depth and width of the network, and achieved significant performance gains by eliminating batch normalization modules in residual networks.

As mentioned above, deep CNN-based SISR architectures have yielded great success but have not fully leveraged the multiscale representation and the intermediate features [11]. Then, Lan et al. [12] explored a model that combines multi-scale residuals with attention mechanism, which can not only extract multi-level features, but also exploits the discriminative information of different channels. A one-shot aggregation network (OAN) [13] employed diverse features with multiple receptive fields by aggregating all previous features into subsequent layers. Inspired by DenseNet [14], Zhang et al. [15] further integrated the dense structure and the residual structure to form a residual dense network (RDN) to exploit the hierarchical features. DenseNet proposed dense connectivity to improve computational efficiency via encouraging feature reuse. However, Huang et al. [16] pointed out that abusing dense connectivity led to redundancies. Each layer did not need to receive information from all the previous layers. This could take up large amounts of memory, which largely restricted the applications on resource-constrained mobile platforms. Hence, Huang et al. [16] introduced CondenseNet, which adopted learned group convolution (LGC) to prune these redundant connections. This produced an efficient, lightweight dense connection network.

Motivated by this, we remove the less important connections from a different viewpoint in this paper. We present a novel method automatic search dense connection network (ASDCN), which utilizes gradient-based neural architecture search (NAS) models [17] to cut superfluous connections automatically. Our entire network has two training procedures. In the first stage, our network selects the right connections through efficient dense connection search. In the second stage, the appropriate structure is trained according to the architecture parameters learned in the first stage. We observe that connection values greater than 0.1 contribute significantly to network performance, and these connections are considered essential to derive the final architecture. Our search space is only for the densely connected joint patterns, without search operations, which can effectively get rid of redundancy. Meanwhile, our proposed method achieves promising results with few parameters. The main contributions of this paper are summarized as follows:

- We introduce a novel lightweight ASDCN model for single image super-resolution, selecting key connection paths effectively, and suppressing redundant information.
- We equip a softmax function to relax the dense connection paths into a continuous space and integrate the architecture search into the model for training. According to the weights of the paths, the appropriate connections are screened out. Selecting the essential features from intermediate layers enables the network to be more compact and efficient.
- Comprehensive experiments on five public benchmark datasets have demonstrated that our derived model achieves comparable performance to the most advanced methods. Our proposed method strikes a trade-off between reconstruction results and model sizes.

## 2. Related Work

### 2.1. Deep CNN-Based Super-Resolution

Due to the emergence of large-scale labeled data and the rapid improvements of GPU [18], CNN-based methods have developed rapidly and obtained state-of-the-art results in a variety of studies [19,20].

Dong et al. [5] first explored a three-layer CNN for SR reconstruction and made a huge improvement compared to traditional methods. Thanks to residual learning, the vanishing gradient problem in deep networks was alleviated. Then, a deeply-recursive convolutional network (DRCN) [21] deployed a deep network by combining residual and recursive ideas, increasing the receptive fields and improving performance. Multi-scale deep super-resolution (MDSR) [10] was devised for simultaneous multi-scale image learning at different magnifications, giving the network sufficient mapping capability and winning the NTIRE2017 challenge. A densely residual Laplacian network (DRLN) [22] utilized dense connections between residual blocks to promote Laplacian attention to assign weights at different scales, resulting in considerable performance gains. Although these networks produce state-of-the-art results, they require large amounts of memory, powerful computing ability and long inference times, and are not suitable for deployment on mobile devices with constrained resources.

Some researchers focused on developing lightweight, but efficient models for SISR [23] without reducing accuracy. A cascading residual network (CARN) [24] constructed a lightweight cascaded residual network through a cascaded scheme with group convolution. Not only did it maintain the most advanced performance but it was faster. An adaptive weighted super-Resolution network (AWSRN) [25] provided an adaptive weighted residual unit to automatically calculate the total residual and initial mapping parameters, achieving better reconstruction quality with lower complexity. In addition, Tian et al. [26] developed a coarse-to-fine CNN for SISR (CFSRCNN), which cascaded multiple hierarchical features to prevent possible training instability and performance degradation, and remarkably improved the computational efficiency. These efficient CNN based models are hand-crafted super-resolution networks. The following section will introduce the NAS-based approaches for SR to achieve optimal performance in an automated manner.

### 2.2. Neural Architecture Search

Neural architecture search (NAS) is an algorithm that automatically learns the appropriate deep neural structure for a specific task with minimal human involvement. The pioneering work of NAS was conducted by [27], who employed the reinforcement learning (RL) method to produce higher accuracy in image classification tasks. Subsequently, evolutionary algorithms [28,29] were introduced to solve NAS problems and achieved considerable classification accuracy on a large scale. Nevertheless, these methods bear hundreds of GPU days. Hence, researchers began to wonder how to reduce the amount of computation and speed up the search for neural structures. An example of this is efficient-NAS (ENAS) [30], which proposed a weight-sharing strategy to improve search efficiency. Compared with NAS, ENAS [30] could shorten GPU computing time by more than 1000 times. Differentiable architecture search (DARTS) [31], another variant of NAS, relaxed a given discrete search space into a continuous space by conducting architecture searches in a differentiable way, and is orders of magnitude faster than the most advanced non-differentiable algorithms. In this paper, we adopt the DARTS algorithm to search the connection pattern of dense connections.

Most of the existing super-resolution models are designed manually and are difficult to compress or fine-tune. At the same time, the neural architecture search algorithm has been highly influential in classification tasks. According to this trend, Chu et al. [32,33] presented fast, accurate, and lightweight SR (FALSR) and multi-objective reinforced evolution in mobile NAS (MoreMNAS), which dealt with super-resolution utilizing a multi-objective method. FALSR-C [32] (a more lightweight version of FALSR) indicated that unwanted features from lower layers could cause problems for high layers to reconstruct SR results. Song et al. [34] built three efficient residual dense blocks to search lightweight SR networks with the evolutionary approach. In addition, these based NAS methods for SR produced brilliant results.

## 3. Proposed Method

### 3.1. Network Architecture

**Figure 1:**
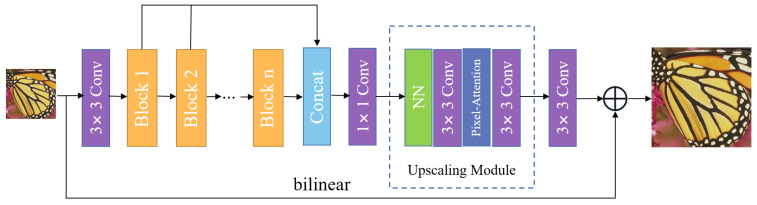
An overview of our ASDCN architecture.

In this section, we introduce our proposed approach. With RDN [15] architecture as the backbone, our proposed network named “automatic search dense connection” (ASDCN) mainly consists of three parts: shallow feature extraction, a nonlinear mapping module with several automatic search dense connection blocks (ASDCBs), and a reconstruction part, as shown in Figure 1. $I_{LR}$ and $I_{SR}$ represent the input raw image and the corresponding high-resolution output of the network, respectively.

Firstly, we leverage one convolution layer to extract low-level features from the original input image, which can be denoted as

(1) $$X_{0}=f_{ext}\left(I_{LR}\right)$$

where $f_{ext}$ is a convolution layer with a kernel size of 3×3 to extract the primitive features from the LR image $I_{LR}$.

Afterwards, the output feature maps $x_{0}$ are fed into the following nonlinear mapping module consisting of a series of stacked multiple ASDCBs to gradually obtain the hierarchical features, which can be represented as

(2) $$X_{t}=f_{B}^{t}\left(X_{t-1}\right)=f_{B}^{t}\left(f_{B}^{t-1}\left(\cdots f_{B}^{0}\left(X_{0}\right)\cdots \right)\right)$$

where $X_{t-1}$ and $X_{t}$ are the input feature maps and output feature maps of the *t*-th ASDCB, respectively. After obtaining the multi-level powerful feature representations, the generated features are concatenated through global feature fusion, which can be denoted as

(3) $$X_{output}=F_{output}\left(Cat\left(X_{1},\cdots ,X_{n}\right)\right)$$

where $F_{output}$ denotes a convolution layer with a kernel size of 1×1. Then, the features $X_{output}$ are up-sampled to the HR image size via an upscaling module. Moreover, two up-sampling modules are required when the scaling factor is ×4. The upscaling module is made up of the nearest neighbor (NN) layer and a pixel attention [35] with two convolution layers interleaved. Each convolution layer is followed by an LReLU [36] activation function while the pixel attention layer contains a 1×1 convolution layer and a sigmoid function. Finally, the interpolated $I_{LR}$ is added to the upsampled $X_{output}$ by global residual connection to obtain the final predicted SR image of the network as follows:

(4) $$I_{SR}=f_{rec}\left(X_{output}\right)+f_{up}\left(I_{LR}\right)$$

where $f_{rec}$ represents the reconstruction module, and $f_{up}$ stands for the bilinear interpolation.

Following previous works [15,24], our network is optimized by $L_{1}$ loss function to measure the difference between the predicted SR image and the ground truth HR image. Given a training set ${\left\{I_{LR}^{i},I_{HR}^{i}\right\}}_{i=1}^{N}$, where *N* is the number of LR-HR training patches, the loss function of our SR network can be expressed as

(5) $$L\left(\Theta \right)=\frac{1}{N}\sum_{k=1}^{N}\left|\right|H_{ASDCN}\left(I_{LR}^{i}\right)-I_{HR}^{i}{\left|\right|}_{1}$$

where $H_{ASDCN}$ denotes our proposed model while Θ indicates the parameters set within it.

### 3.2. Automatic Search Dense Connection Module

This section describes how to search for dense connections using the gradient-based NAS method. As is common knowldge, DenseNet allows feature maps from all previous layers entering into the subsequent layers to make the most use of the features. However, there remain some redundant connections which will affect the efficiency of the network. It is difficult to determine which remaining features are unnecessary. To this end, we devise an adaptive structure to prune unimportant connections while retaining useful ones during training.

**Figure 2:**
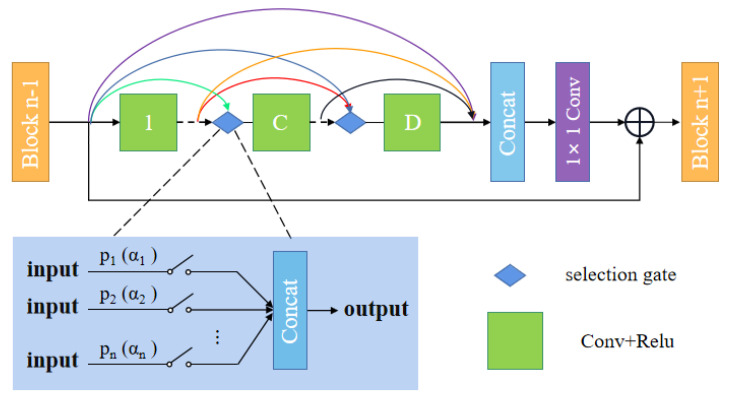
The automatic dense connection module.

Figure 2 shows our proposed automatic search dense connection block (ASDCB). Our search space is composed of dense connections between distinct layers of each block. The key idea of the proposed method is to relax the discrete densely connected space into a continuous representation, which allows us to choose the candidate paths with significant contributions in a differentiable manner. To this end, we exploit a softmax function for the continuous relaxation of the search space. We assign an initial probability parameter to each path of dense connection between various layers in one block. During the search process, the probability parameters are optimized. We further sort all the candidate paths, which can help us to screen out the path with a more outstanding contribution and eliminate the path with less of a contribution. By doing so, superior candidate architectures can be searched for further experiments to obtain better results.

We relax dense connections into continuous representations and assign an architecture parameter α to each output path of the layer. Let *o* be the set of candidate connection paths, and $\alpha_{o}^{\left(i,j\right)}$ be the weight of each output path of the layer. We employ a softmax function to compute the probability of each input path over all paths in one layer as follows:

(6) $$P\left(\alpha_{o}^{\left(i,j\right)}\right)=\frac{\exp(\alpha_{o}^{\left(i,j\right)})}{\sum_{o^{\prime }\in O}\exp\left(\alpha_{o^{\prime }}^{\left(i,j\right)}\right)}$$

The output of each layer is computed based on all of its previous layer in one block, and can be expressed as

(7) $$x_{n+1}=\sum_{o\in O}P\left(\alpha_{o}^{\left(i,j\right)}\right)\cdot o\left(x_{n}\right)$$

where $x_{i}$ represents the input tensor of layer *n*, and *o* stands for the convolution operation. Hence, the architecture search can be treated as an optimization problem for a set of continuous variables $\alpha =\alpha_{o}^{\left(i,j\right)}$.

Through an automatic searching strategy, the connection paths that contribute most are selected, while other paths are discarded. Then, the final architecture is derived from the learned parameters. The search process is described in the next section.

### 3.3. Search Procedure

Based on the continuous relaxation of the search space, we can leverage the gradient descent strategy to optimize the architecture parameters and network weights jointly. Let α be the parameters of the proposed module and ω be the parameters of the whole network, and the training process can be described as:

(8) $$\min_{\alpha }L_{val}\left(\omega^{*}\left(\alpha \right),\alpha \right)$$

(9) $$s.t.\;\omega^{*}\left(\alpha \right)=\arg\min_{\omega }L_{train}\left(\omega ,\alpha \right)$$

where $L_{train}$ and $L_{val}$ indicate the training and validation loss, respectively.

We aim to jointly optimize the architecture parameters α and weights ω of the network, so that the architecture finds the minimum training and validation loss. First, we optimize the network weights ω by descending $\nabla_{\omega }L_{train}\left(\omega ,\alpha \right)$ for enough epochs to warm up on the training dataset. After warming up ω, we update the architecture parameters by descending $\nabla_{\omega }L_{train}\left(\omega^{*}\left(\alpha \right),\alpha \right)$ into validation datasets. The architecture parameters α are randomly initialized. Then, $P(\alpha_{o}^{\left(i,j\right)})$ is defined as the importance of all input paths. A particular layer can be calculated by Formula (6) to determine which paths are retained. We set a threshold of 0.1 (the paths will be discarded when $P(\alpha_{o}^{\left(i,j\right)})<0.1$). According to these learned parameters, we choose the most appropriate candidate paths to derive the final architecture for experiments. The whole search procedure is shown in Algorithm 1.

Our proposed algorithm shows that dense connections are not always the best way to transmit information. Features with small contributions from lower layers can cause problems in reconstructing super-resolution results at the high-level layer. We discard input paths that contribute little to each layer in the block during the search process, while reserving input paths with high weights. Compared with the pruning weights in a pre-trained network, our method is lighter and more efficient. It not only restricts the front-end redundancy of DenseNet and reduces the number of parameters, but also achieves competitive performance.
**Algorithm 1** Training process.**Require:** epoch, architecture parameter $\alpha^{\left(i,j\right)}$  1:         network weight ω  2:initialize α and ω  3:**for** epoch ≤ 20 **do**  4:   update ω  5:**end for**  6:**for** not converged **do**  7:   update 1 $\alpha ⟵\nabla_{\alpha }L_{val}\left(\omega -\varepsilon \nabla_{\omega }L_{train}\left(\omega ,\alpha \right),\alpha \right)$  8:   update 2 $\omega ⟵\nabla_{\omega }L_{train}\left(\omega ,\alpha \right)$  9:**end for**10:Derive the final architecture and retrain.

## 4. Experiments

### 4.1. Datasets and Metrics

In order to make fair comparisons with the state-of-the-art SR algorithms, we follow previous works. DIV2K [37] is a recent high-resolution dataset, which includes 800 training images, 100 validation images, and 100 test images. We adopt 800 pairs of LR and HR training images from DIV2K to train our model, and the LR images are obtained via the bicubic downsampling of the corresponding HR images. In addition, Set5 [38] is adopted for validation after each epoch. In the testing phase, we employ several public benchmark datasets (Set14 [39], BSD100 [40], Urban100 [41], and Manga109 [42]) to evaluate the performance of our proposed algorithm under three upscaling factors (×2, ×3, and ×4). The peak signal-to-noise ratio (PSNR) and the structural similarity index (SSIM) [43] on the Y channel of transformed YCbCr space are treated as quantitative evaluation metrics.

Given a ground-truth image $I^{HR}$ and a predicted image $I^{SR}$, the PSNR is formulated as:

(10) $$PSNR\left(I^{HR},I^{SR}\right)=10log_{10}\left(\frac{Max_{I}^{2}}{MSE}\right)$$

where

(11) $$MSE=\frac{1}{H\times W}\sum_{i=1}^{H}\sum_{j=1}^{W}{\left(I^{HR}\left(i,j\right)-I^{SR}\left(i,j\right)\right)}^{2}$$

$Max_{I}$ is the maximum pixel value of an image. *H* and *W* are the height and width of a image, respectively. SSIM is defined as:

(12) $$SSIM\left(I^{HR},I^{SR}\right)=l\left(I^{HR},I^{SR}\right)c\left(I^{HR},I^{SR}\right)s\left(I^{HR},I^{SR}\right),$$

where

(13) $$\left\{\begin{array}{cc} l\left(I^{HR},I^{SR}\right)=\frac{2\mu_{I^{HR}}\mu_{I^{SR}}+C_{1}}{\mu_{I^{HR}}^{2}+\mu_{I^{SR}}^{2}+C_{1}} & \\ c\left(I^{HR},I^{SR}\right)=\frac{2\sigma_{I^{HR}}\sigma_{I^{SR}}+C_{2}}{\sigma_{I^{HR}}^{2}+\sigma_{I^{SR}}^{2}+C_{2}} & \\ s\left(I^{HR},I^{SR}\right)=\frac{2\sigma_{I^{HR}I^{SR}}+C_{3}}{\sigma_{I^{HR}}\sigma_{I^{SR}}+C_{3}} \end{array}\right.$$

$\mu_{I^{HR}}$, $\sigma_{I^{HR}}$, and $\sigma_{I^{HR}I^{SR}}$ are the mean values, variance, and covariance of an image, respectively. $C_{1}$, $C_{2}$ and $C_{3}$ are set to positive constants to avoid instability when the denominator is close to zero.

Moreover, we calculate Multi-Adds and the number of parameters to assess the complexity of our model. Multi-Adds are computed on HR images with a spatial resolution of 720p at all scales.

### 4.2. Implementation Details

The whole training process is split into the searching and retraining phases. The MATLAB function is applied to the bicubic downsampling of the counterpart HR images to obtain the corresponding LR images. We randomly crop image patches with the size of 96×96 for the searching phase (144×144 for the retraining phase) from the LR images, and 16 patches are utilized as input for each training minibatch. Data augmentation is conducted by random rotations of $90^{\circ }$, $180^{\circ }$, $270^{\circ }$, and horizontal flips for each training iteration. The searching stage and retraining stage contain 200 and 1000 epochs, respectively. We update only network weights for the first 20 epochs in the searching stage. Then, the architecture parameters are updated using the early stop strategy. We set 1000 iterations as an epoch.

In our model, except for feature fusion parts equipped with 1×1 convolution, the other parts all use a 3×3 convolution layer. Furthermore, we employ a padding strategy to keep the size of the output feature maps the same for each layer. Our ASDCN model consists of ten automatic search dense connection blocks (ASDCBs). Each block has six 3×3 convolution layers and a 1×1 convolution layer. A convolution layer with a kernel size of 1×1 is employed to match the channels and preserve more useful information. The channel of the intermediate layer within each block is fixed to 16.

Our network is optimized using the Adam [44] optimizer by setting $\beta_{1}$=0.9 and $\beta_{1}$=0.999. We leverage cosine annealing to reduce the learning rate. The maximum learning rate is initialized as $10^{-4}$, and the target minimum learning rate is fixed as $10^{-6}$. The learning rate of the architecture parameter is set to 0.002. Additionally, different from reference [45] using dynamic differential evolution, for the hyperparameters of network search, the best ones are selected through repeated experiments on the search process. For the hyperparameters of the network structure, several combinations of the number of blocks and convolutional layers are repeatedly tested to choose the optimal ones under a specific parameter amount. The other hyperparameters are provided by the reference image super-resolution methods without trial and error. Our model is trained using the PyTorch framework with an NVIDIA RTX 2080Ti GPU.

### 4.3. Ablation Study

#### 4.3.1. Comparison with RDN with the Same Setup

In this section, we make a comparison with RDN [15] under the same setup. We employ the same training dataset to train these two models. The two models have six blocks, and the channel number of intermediate layers is set to 16. The experimental results with a scaling factor of ×2 in five available datasets are shown in Table 1. It can be seen that our proposed algorithm is slightly better than RDN with PSNR and SSIM, but Multi-Adds and the number of parameters are significantly fewer than RDN. This indicates that dense connections are not always the best way to transmit information. There are still redundant connections, and it is not necessary to feed each previous layer into the next layer. Our designed strategy for automatically searching dense connection patterns can selectively use the essential features from previous layers, which can reduce redundancy and improve network efficiency. It is further shown that the adaptive dense network improvement method can obtain a lightweight model with comparable performance.

#### 4.3.2. Searched Architectures

**Figure 3:**
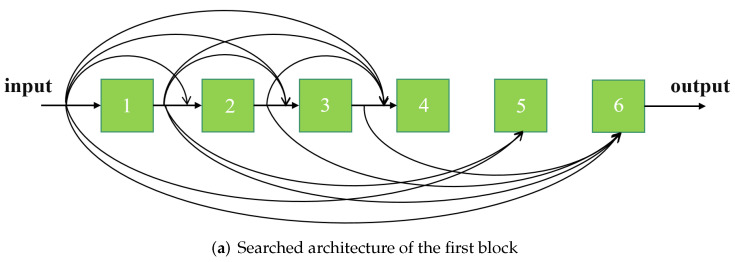

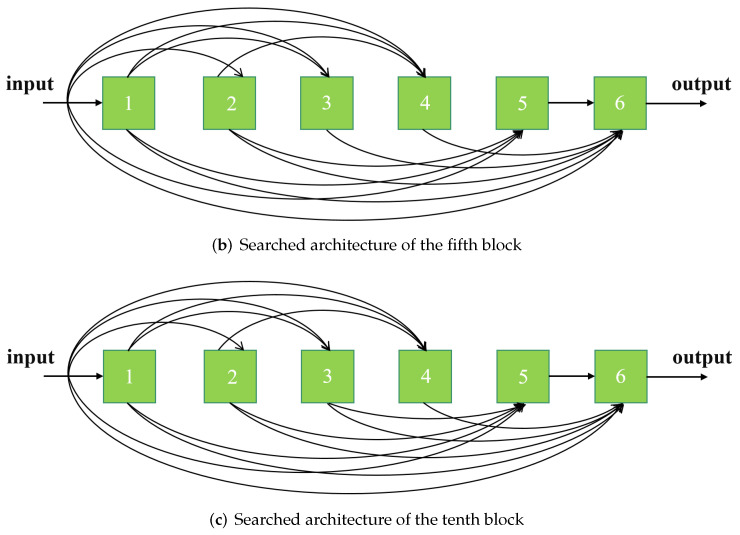
The searched architectures of different blocks.

This section shows the internal connection patterns for the first, fifth, and tenth ASDCB architecture. It is clear from Figure 3 that not every intermediate layer accepts the output of each previous layer. Thus, the adaptive selection of connection paths can effectively reduce redundancy and boost the efficiency of the network without degrading performance.

### 4.4. Comparison with State-of-the-Art Methods

To demonstrate the effectiveness of our proposed architecture, we compared ASDCN with other state-of-the-art models, including SRCNN [5], VDSR [6], DRCN [21], Laplacian pyramid super-resolution network (LapSRN) [3], CARN-M [24], MoreMNAS-A [33], FALSR-C [32], AWSRN-S [25], efficient super-resolution network (ESRN-V) [34], multiscale attention dual network (MADNet-L1) [12], OAN-S [13], and weighted multi-scale residual network (WMRN) [4]. These are lightweight models within the number of parameters 2.0 M or Multi-Adds 100 G. Parameters (space complexity) and Multi-Adds (time complexity) are used to reflect the complexity of our model. The quantitative comparisons for ×2, ×3, and ×4 are depicted in Table 2 on the five datasets.

As can be seen from Table 2, the proposed model is superior to the most advanced models at different scaling factors with less than 2 M parameters. Under comparable computational complexity, our ASDCN achieves higher PSNR values than hand-designed CARN-M. Compared with the manually constructed WMRN, our derived architecture obtains a better reconstruction result, but the number of parameters and Multi-Adds are reduced by about 53%. Compared with MADNet-L1, ASDCN achieves higher reconstruction accuracy with fewer parameters and Multi-Add. Moreover, our searched model also outperforms the three most advanced NAS-based approaches (FALSR-C, ESRN-V, and MoreMNAS-A) for ×2 SR on all the benchmark datasets. Specifically, MoreMNAS-A has three times as large as ours in terms of parameters and Multi-Adds, but our PSNR value has a considerable margin of 0.63 dB PSNR that of Urban100.

In addition, Table 2 also provides the complexity (the amount of parameters and Muti-Adds) of the different models for a more intuitive comparison. The parameters and Muti-Adds of SRCNN and VDSR do not change at the all scales because the bicubic interpolation images are required as input, and other methods have inconsistent changes. Since our model has relatively few parameters and Multi-Adds is also not high, it is a lightweight model.

**Figure 4:**
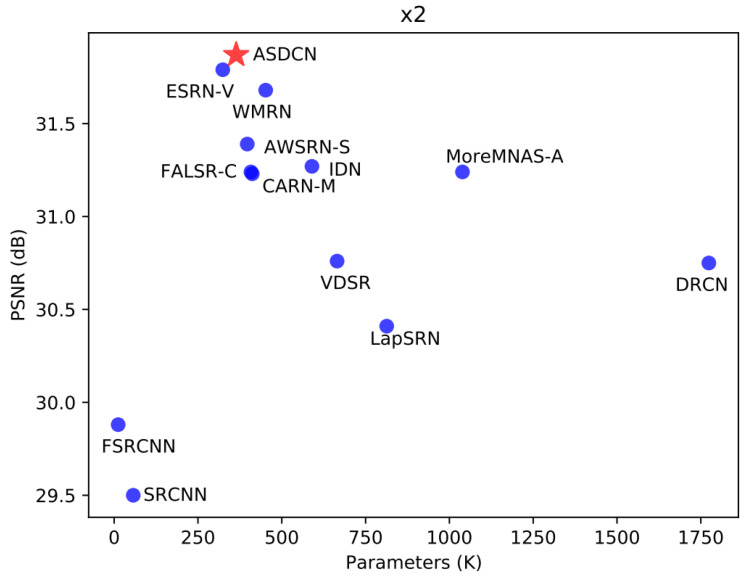
Comparison of the performance and parameters between our ASDCN model and other models on Urban100 with a scale factor of 2.

Figure 4 further compares the number of parameters and performance of different approaches. The results show that our method exceeds other methods in both parameters and performance, which fully proves that we have achieved a better balance between model size and performance.

**Figure 5:**
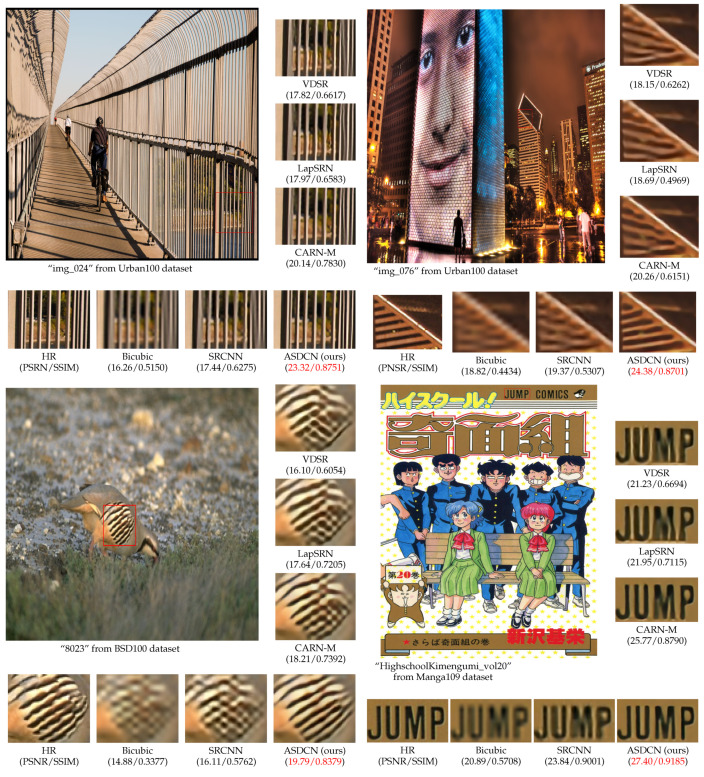
Visual comparison of ×4 super-resolution images on the Urban100, BSD100, and Manga109 datasets. The best results are highlighted by red.

In addition to quantitative evaluation, we also visually compare our model with other models. Figure 5 shows the subjective visual quality on three datasets with ×4 the upscaling factor. For the “img_024” and “img_076” from Urban100, only our method can restore the correct lines and suppress the distortions, whereas other methods cannot reconstruct the proper structure. For “HighschoolKimengumi_vol20” from Manga109, our network has more precise texture information and edges with less blurring and artifacts. Furthermore, for “0823” from BSD100, we can also notice that our derived architecture produces the best reconstruction effect, whereas VDSR has checkerboard artifacts, and CARN-M has more blurring and noise.

### 4.5. Visualization on Real-World Images

To verify the effectiveness and robustness of the proposed algorithm, we further compare it with other methods on real-world images. There is no high-quality ground truth in these cases, and the degradation model is unknown.

**Figure 6:**
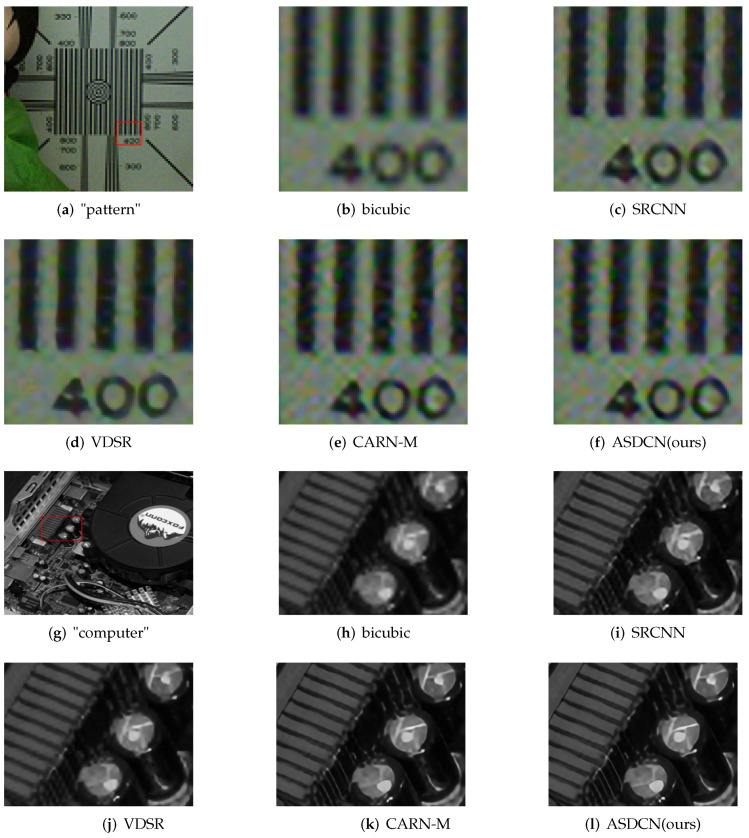
Visual comparison with a scale factor ×4 on real-world images.

Figure 6 shows that our method can precisely recover more image details and more apparent contours. The better perceptual quality further indicates that our derived architecture can search for more convincing SR models.

## 5. Conclusions

In this paper, we propose a framework for automatically searching dense connection modules for single image super-resolution. The NAS-based method used to search dense connection paths can adaptively select the key connection paths and effectively reduce the redundant information of the network. Moreover, it is more efficient than manual pruning. The lightweight image super-resolution is realized by efficient residual dense connection blocks and multi-layer information fusion. Extensive quantitative and qualitative experiments demonstrate that our derived model is superior to most state-of-the-art approaches with comparable parameters and Multi-Adds.

Our model only searches the intermediate nodes of the dense block, and only searches which pre-nodes need to be used and which can be discarded. Our future work will extend to search the operation of the model, and even search the block level of the entire model synchronously. Of course, we can also study non-NAS methods later, such as permuting and combining more basic operations (multiplication, addition, etc.).

## Figures and Tables

**Table 1:** Comparison with RDN on five benchmark datasets. All the other settings are strictly the same. The best performance is highlighted by red.

Method	Params	Multi-Adds	Set5	Set10	BSD100	Urban100	Manga109
	(K)	(G)	PSNR/SSIM	PSNR/SSIM	PSNR/SSIM	PSNR/SSIM	PSNR/SSIM
RDN	520	119.9	37.90/0.9601	33.45/0.9165	32.12/0.8990	31.87/0.9259	38.28/0.9764
ASDCN (ours)	364	83.8	37.91/0.9603	33.48/0.9176	32.12/0.8990	31.87/0.9261	38.30/0.9765

**Table 2:** Quantitative results of several state-of-the-art SR models at scaling factors of ×2, ×3 and ×4 (average PSNR/SSIM). The best performance is highlighted by red.

Method	Scale	Params	Multi-Adds	Set5	Set10	BSD100	Urban100	Manga109
		(K)	(G)	PSNR/SSIM	PSNR/SSIM	PSNR/SSIM	PSNR/SSIM	PSNR/SSIM
SRCNN [5]	×2	57	52.7	36.66/0.9542	32.42/0.9063	31.36/0.8879	29.50/0.8946	35.60/0.9663
VDSR [6]	×2	665	612.6	37.53/0.9587	33.03/0.9124	31.90/0.8960	30.76/0.9140	37.22/0.9729
LapSRN [3]	×2	813	29.9	37.52/0.9590	33.08/0.9130	31.80/0.8950	30.41/0.9100	37.27/0.9740
IDN [23]	×2	590	174.1	37.83/0.9600	33.30/0.9148	32.08/0.8950	31.27/0.9196	-
CARN-M [24]	×2	412	91.2	37.53/0.9583	33.26/0.9141	31.92/0.8960	31.23/0.9193	-
MoreMNAS-A [33]	×2	1039	238.6	37.63/0.9584	33.23/0.9138	31.95/0.8961	31.24/0.9187	-
FALSR-C [32]	×2	408	93.7	37.66/0.9586	33.26/0.9140	31.96/0.8965	31.24/0.9187	-
AWSRN-S [25]	×2	397	91.2	37.75/0.9596	33.31/0.9151	32.00/0.8974	31.39/0.9207	37.90/0.9755
ESRN-V [34]	×2	324	73.4	37.85/0.9600	33.42/0.9161	32.10/0.8987	31.79/0.9248	-
MADNet-L1 [12]	×2	878	187.1	37.85/0.9600	33.38/0.9161	32.04/0.8979	31.62/0.9233	-
OAN-S [13]	×2	450	104.9	37.85/0.9600	33.41/0.9162	32.06/0.8983	31.61/0.9230	38.16/0.9761
WMRN [4]	×2	452	103	37.83/0.9599	33.41/0.9162	32.08/0.8984	31.68/0.9241	38.27/0.9763
ASDCN(ours)	×2	364	83.8	37.91/0.9603	33.48/0.9176	32.12/0.8990	31.87/0.9261	38.30/0.9765
SRCNN [5]	×3	57	52.7	32.75/0.9090	29.28/0.8209	28.41/0.7863	26.24/0.7989	30.59/0.9107
VDSR [6]	×3	665	612.6	33.66/0.9213	29.77/0.8314	28.82/0.7976	27.14/0.8279	32.01/0.9310
CARN-M [24]	×3	412	46.1	33.99/0.9236	30.08/0.8367	28.91/0.8000	26.86/0.8263	-
IDN [23]	×2	590	105.6	34.11/0.9253	29.99/0.8354	28.95/0.8013	27.42/0.8359	-
AWSRN-S [25]	×3	447	48.6	34.02/0.9240	30.09/0.8376	28.92/0.8009	27.57/0.8391	32.82/0.9393
ESRN-V [34]	×3	324	36.2	34.23/0.9262	30.27/0.8400	29.03/0.8039	27.95/0.8481	-
MADNet-L1 [12]	×3	930	88.4	34.16/0.9253	30.21/0.8398	28.98/0.8023	27.77/0.8439	-
OAN-S [13]	×3	490	51.2	34.17/0.9255	30.20/0.8395	28.99/0.8023	27.80/0.8438	33.06/0.9144
WMRN [4]	×3	556	57	34.11/0.9251	30.17/0.8390	28.98/0.8021	27.80/0.8448	33.07/0.9413
ASDCN(ours)	×3	364	37.28	34.27/0.9266	30.27/0.8413	29.06/0.8041	28.03/0.8499	33.28/0.9430
SRCNN [5]	×4	57	52.7	30.48/0.8628	27.49/0.7503	26.90/0.7101	24.52/0.7221	27.66/0.8505
VDSR [6]	×4	665	612.6	31.35/0.8838	28.01/0.7674	27.29/0.7251	25.18/0.7524	28.83/0.8809
LapSRN [3]	×4	813	149.4	31.54/0.8850	28.19/0.7720	27.32/0.7280	25.21/0.7560	29.09/0.8845
IDN [23]	×4	590	81.8	31.82/0.8903	28.25/0.7730	27.41/0.7297	25.41/0.7632	-
CARN-M [24]	×4	412	32.5	31.92/0.8903	28.42/0.7762	27.44/0.7304	25.63/0.7688	-
AWSRN-S [25]	×4	588	33.7	31.77/0.8893	28.35/0.7761	27.41/0.7304	25.56/0.7678	29.74/0.8982
ESRN-V [34]	×4	324	20.7	31.99/0.8919	28.49/0.7779	27.50/0.7331	25.87/0.7782	-
MADNet-L1 [12]	×4	1002	54.1	31.95/0.8917	28.44/0.7780	27.47/0.7327	25.76/0.7746	-
OAN-S [13]	×4	520	42.5	31.99/0.8926	28.49/0.7975	27.49/0.7332	25.81/0.7760	30.10/0.9036
WMRN [4]	×4	536	45.7	32.00/0.8952	28.47/0.7786	27.49/0.7328	25.89/0.7789	30.11/0.9040
ASDCN(ours)	×4	375	21.59	32.06/0.8937	28.53/0.7806	27.54/0.7351	25.98/0.7831	30.23/0.9063

## Data Availability

Not applicable.
